# RERT: A Novel Regression Tree Approach to Predict Extrauterine Disease in Endometrial Carcinoma Patients

**DOI:** 10.1038/s41598-017-11104-4

**Published:** 2017-09-05

**Authors:** Marika Vezzoli, Antonella Ravaggi, Laura Zanotti, Rebecca Angelica Miscioscia, Eliana Bignotti, Monica Ragnoli, Angela Gambino, Giuseppina Ruggeri, Stefano Calza, Enrico Sartori, Franco Odicino

**Affiliations:** 1 Department of Molecular and Translational Medicine, Unit of Biostatistics, University of Brescia, Brescia, Italy; 2 “Angelo Nocivelli” Institute of Molecular Medicine, Division of Obstetrics and Gynecology, University of Brescia, Brescia, Italy; 3 Department of Obstetrics and Gynecology, University of Brescia, Brescia, Italy; 4 Division of Obstetrics and Gynecology, ASST Spedali Civili of Brescia, Brescia, Italy; 5 Laboratory Analysis, ASST Spedali Civili of Brescia, Brescia, Italy; 6 Department of Medical Epidemiology and Biostatistics, Karolinska Institutet, Stockholm, Sweden

## Abstract

Some aspects of endometrial cancer (EC) preoperative work-up are still controversial, and debatable are the roles played by lymphadenectomy and radical surgery. Proper preoperative EC staging can help design a tailored surgical treatment, and this study aims to propose a new algorithm able to predict extrauterine disease diffusion. 293 EC patients were consecutively enrolled, and age, BMI, children’s number, menopausal status, contraception, hormone replacement therapy, hypertension, histological grading, clinical stage, and serum HE4 and CA125 values were preoperatively evaluated. In order to identify before surgery the most important variables able to classify EC patients based on FIGO stage, we adopted a new statistical approach consisting of two-steps: 1) Random Forest with its relative variable importance; 2) a novel algorithm able to select the most representative Regression Tree (RERT) from an ensemble method. RERT, built on the above mentioned variables, provided a sensitivity, specificity, NPV and PPV of 90%, 76%, 94% and 65% respectively, in predicting FIGO stage > I. Notably, RERT outperformed the prediction ability of HE4, CA125, Logistic Regression and single cross-validated Regression Tree. Such algorithm has great potential, since it better identifies the true early-stage patients, thus providing concrete support in the decisional process about therapeutic options to be performed.

## Introduction

Endometrial cancer (EC) represents the most common gynecological carcinoma in the Western world. In the United States it accounts for 7% of all female malignant neoplasms with 60,050 estimated new cases and 10,470 estimated cancer deaths (4% of the total among women) in 2016^1^. Most of the patients (71%) are diagnosed at an early stage of disease (FIGO stage I) and this results in a good prognosis, with a five-year survival rate of 90%^2^.

EC can be classified into two types, the type I (endometrioid histotype) and the type II (serous, clear cell, undifferentiated histotypes), characterized by different clinical, pathological, and biological features^3^.

While early stage type I EC is usually treated through extra-fascial hysterectomy with bilateral salpingo-oophorectomy, the optimal surgical management for more advanced stage type I disease is not yet univocally defined. The preoperative work-up and surgical approaches are still controversial, especially with regards to the role played by lymphadenectomy (type and extent) and radical surgery^4–7^.

Furthermore, not all patients preoperatively defined as “clinically early stage” (FIGO stage I) are “truly early stage”, since at least 20% of these patients are definitively post surgically staged as advanced (FIGO stage II-IV). Unfortunately, conventional preoperative assessment, based on imaging techniques (Magnetic Resonance Imaging, Computed Tomography, Transvaginal Sonography) and endometrial biopsy, are not able to systematically identify the extrauterine diffusion of disease and thus to correctly stratify between “truly early stage” vs “truly advanced stage” patients^8^.

Bethinking the discordance between clinical and surgical/pathological evaluation, the use of a preoperative factor, such as serum biomarkers assessment, could provide a standardized, reliable and reproducible parameter to identify patients whom would more likely benefit from a more aggressive surgery. An optimal preoperative evaluation could allow a tailored surgical treatment (extra-fascial hysterectomy vs radical hysterectomy – pelvic and/or paraaortic lymphadenectomy), minimizing both morbidity and costs.

Among different serum biomarkers so far investigated in EC, serum CA125 (sCA125) and Human epididymis protein 4 (sHE4) have been mostly reported in literature. In particular, elevated preoperative sCA125 has been usually associated with advanced FIGO stage and lymph node metastasis, even though the sensitivity in predicting extrauterine disease is controversial^9–11^.

In addition, sHE4 was recently found significantly associated with deeper myometrial invasion, higher histological grade, lymph node metastasis, and advanced FIGO stage^12–14^, suggesting its possible application in the preoperative assessment of EC patients.

Most clinical research deals with traditional parametric statistical methods, which however expose to the risk of seriously biased results due to the nature of the variables under inspection. In this framework, a significant methodological improvement comes from non-parametric approaches, specifically Regression Trees and related ensemble methods (among others)^15–18^. These techniques are able to detect complex and non-linear interactions between response variable and covariates of heterogeneous nature (qualitative and quantitative) and are robust in case of missing values and multicollinearity in the data^15^.

The present study aims to preoperatively predict the surgical/pathological stage of the disease in a large cohort of EC patients, using a novel statistical approach, called REpresentative Regression Tree (RERT), combined with Random Forest^19^ and its relative variable importance, in order to identify the main drivers (HE4 and CA125 serum biomarkers, together with other clinical and pathological variables) impacting on FIGO Stage. The new procedure proposed in this paper is not a breakthrough of the literature, but a generalization of the well-known Regression Trees whose main advantage is to provide more stable and accurate predictions than the canonical data mining methods.

## Results

A total of 370 patients with EC were enrolled in this study. Lymph node sampling or dissection was not performed on 33 patients, in consideration of the presence of comorbidities or according to clinical evaluation (very low risk EC). Hence, due to the lack of a full surgical staging procedure, these patients were excluded from further biomarker analysis. Differently, even though 10 patients with FIGO stage ≥ III were not subjected to lymphadenectomy, they were included anyway in the study because lymph node status showed no impact on their advanced FIGO stage.

Thirty-seven patients were not considered because affected by synchronous endometrial and ovarian cancer. Another 7 patients were excluded because of renal failure with creatinine >1.5 mg/dl that is a well-recognized cause of sHE4 increase in non-oncologic patients^20, 21^.

Therefore, sHE4 and sCA125 evaluation was performed in a total of 293 EC patients. Patients’ clinico-pathological characteristics are represented in Table 1. The median levels of sHE4 and sCA125 and other statistic data (range, 95^th^ percentile and *p-values*) are reported in Table 1, with respect to the other variables in the dataset. Both markers exhibited a significant association with clinical and surgical FIGO stage, myometrial and cervical invasion, ovarian metastases, lymph node status, lymphovascular invasion, positive peritoneal cytology. Only sHE4 was significantly associated with age, number of children, menopause status, hormone replacement therapy (HRT), hypertension, and grading.

**Table 1:** Preoperative sCA125 and sHE4 levels in all 293 EC patients divided according to clinicopathological features

Variables	n.	HE4 pmol/L				CA125 U/mL			
		Median	Range	95th perc	*p-value*	Median	Range	95th perc	*p-value*
**Age** (**years**)									
<50	22	49.70	24,70–176	148.87		20.15	5,00–433,00	373.48	
≥50	271	78.00	6,5–653	312.00	***0***.***0247***	17.25	2,40–2922,00	120.11	*0*.*4134*
**BMI**									
<18.5	5	97.50	54,00–649,00	543.76		18.00	12,70–60,00	58.60	
18.5–25	102	76.75	6,50–639,40	410.22		19.50	3,90–961,00	198.00	
25–30	94	69.35	24,70–410,00	262.66		15.20	2,40–165,20	67.10	
≥30	78	83.95	32,20–653,00	316.5	*0*.*2106*	17.95	3,30–2922,00	197.76	*0*.*0927*
Missing values	14								
**Number of children**									
<4	275	76.60	6,50–653,00	279.53		17.25	2,40–2922,00	141.89	
≥4	18	87.70	45,00–649,00	393.06	***0***.***0476***	18.95	3,7–107,50	61.17	*0*.*8244*
**Menopause status**									
No	35	52.20	24,70–541,90	165.92		16.80	5,00–433,00	282.30	
Yes	257	79.40	6,50–653,00	312.00	***0***.***0087***	17.75	2,40–2922,00	120.05	*0*.*6322*
Missing values	1								
**Contraception**									
No	193	77.00	24,70–653,00	312.00		18.15	2,40–2922,00	155.74	
Yes	13	60.20	32,20–176,00	159.86	*0*.*0653*	15.00	6,2–433,00	316.00	*0*.*7334*
Missing values	87								
**HRT**									
No	223	76.70	6,50–653,00	311.23		18.25	3,30–2922,00	172.61	
Yes	41	64.20	19,40–410,00	175.00	***0***.***0480***	16.90	2,40–118,00	71.70	*0*.*3554*
Missing values	29								
**Hypertension**									
No	143	67.00	6,50–653,00	223.69		17.80	3,30–2922,00	162.54	
Yes	150	85.90	30,80–639,40	312.00	***0***.***0010***	17.70	2,40–497,40	96.50	*0*.*3873*
**Grading from biopsy**									
Hyperplasia	6	46.90	30,80–53,30	52.77		10.65	7,90–15,40	14.50	
G1	82	67.65	24,70–526,00	219.18		17.25	5,20–263,00	77.76	
G2	91	85.90	19,40–649,00	311.50		20.60	3,30–497,40	120.11	
G3	87	78.00	6,50–653,00	308.61	***0***.***0013***	16.80	4,60–2922,00	186.20	*0*.*0664*
Missing values	27								
**Clinical stage** (**presurgical**)									
Early (FIGO ≤I)	246	72.95	6,50–639,40	294.10		17.00	2,40–263,00	73.14	
Advanced (FIGO > I)	44	103.55	41,00–653,00	531.10	<***0***.***001***	36.55	5,90–2922,00	487.74	<***0***.***001***
Missing values	3								
**Surgical FIGO stage**									
I	194	66.10	6,50–346,70	197.40		15.90	2,40–238,00	58.12	
>I	99	107.40	34,5–653,00	526.60	<***0***.***001***	25.00	3,90–2922,00	299.90	***0***.***0063***
**Histotype**									
Non endometrioid	39	71.00	31,70–653,00	424.74		26.00	4,70–2922,00	220.07	
Endometrioid	254	76.80	6,50–649,00	301.96	*0*.*5963*	17.20	2,40–961,00	106.60	*0*.*0629*
**Surgical Grading**									
G1	68	61.95	24,70–304,30	152.70		15.90	2,40–238,00	51.16	
G2	127	84.50	6,50–649,00	321.69		18.10	3,30–497,40	120.14	
G3	98	84.25	31,70–653,00	415.22	<***0***.***001***	19.00	3,90–2922,00	197.76	*0*.*1543*
**Myometrial invasion**									
M0	37	48.90	6,50–138,90	103.12		15.20	4,70–71,20	42.25	
M1	107	66.20	31,70–653,00	215.68		15.50	2,40–2922,00	54.40	
M2	149	94.00	19,40–649,00	412.10	<***0***.***001***	22.00	3,70–961,00	188.00	<***0***.***001***
**Extension to cervix**									
No	208	67.60	6,50–653,00	219.86		17.00	2,40–2922,00	95.20	
Yes	85	94.70	34,50–649,00	412.80	<***0***.***001***	20.50	4,60–961,00	190.76	***0***.***0104***
**Ovarian metastases**									
No	260	72.95	6,50–649,00	226.61		16.90	2,40–389,00	84.69	
Yes	31	100.30	50,50–653,00	536.95	<***0***.***001***	40.00	7,00–2922,00	729.20	<***0***.***001***
Missing values	2								
**Lymph nodes status**									
Negative	243	70.70	6,50–649,00	223.19		15.90	2,40–238,00	59.91	
Positive	40	108.40	50,50–532,00	410.75	<***0***.***001***	38.25	4,60–961,00	394.42	<***0***.***001***
Missing values	10								
**Lymphovascular invasion**									
Absent	128	62.10	6,50–347,90	159.37		14.30	2,40–238,00	57.01	
Present	156	90.05	19,40–653,00	416.38	<***0***.***001***	22.90	3,90–2922,00	201.57	<***0***.***001***
Missing values	9								
**Positive peritoneal cytology**									
No	258	74.65	6,50–649,00	275.30		17.00	2,40–497,40	109.60	
Yes	29	110.00	50,50–653,00	537.94	<***0***.***001***	32.00	7,00–2922,00	692.60	<***0***.***001***
Missing values	6								

*p-values* were computed using non-parametric Wilcoxon-Mann-Whitney or Kruskal-Wallis test. In bold *p-values* < *0*.*05*. Missing values are not considered in the test procedure.

BMI = Body Mass Index; HRT = Hormone Replacement Therapy.

A linear positive relationship between the two markers was present, as shown by the Pearson’s correlation coefficient (*ρ* = 0.4872, *p-values* < 0.001).

Among all preoperatively available clinical/pathological variables, only sHE4 (*p-value* < 0.001) and sCA125 (*p-value* < 0.001) (Table S2 in Supplementary Information, page 3), pre-surgical clinical stage (*p-value* < 0.001) and histological grading from biopsy (*p-value* = 0.0096) (Table S3 in Supplementary Information, page 3) were significantly and positively associated with FIGO stage > I.

**Figure 1:**
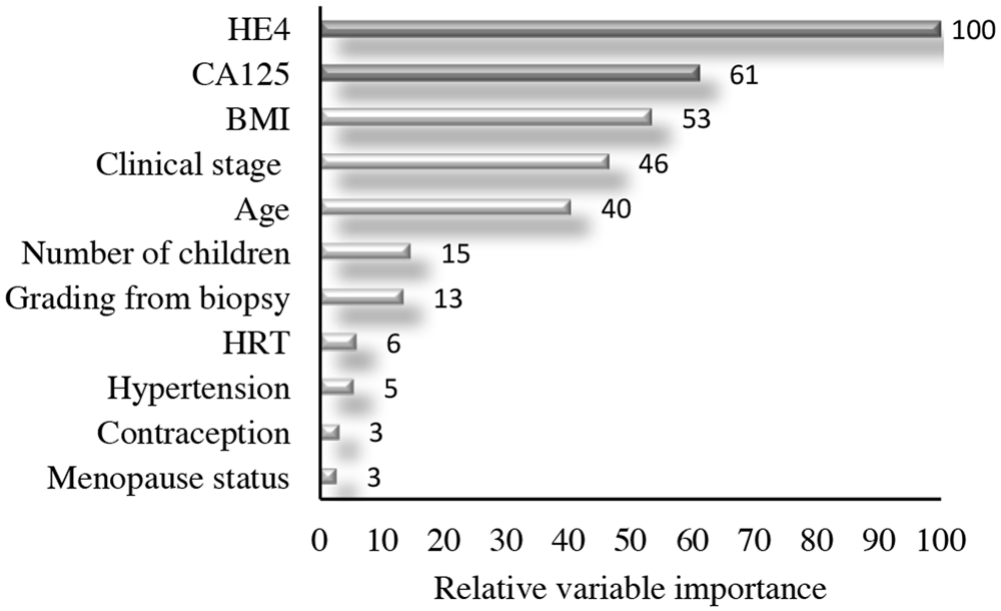
Relative variable importance measure. Total Decrease in Node Impurity- obtained by Random Forests on all 293 EC patients.

In order to identify the main drivers in surgical FIGO stage prediction, we ran the Random Forest, growing 10,000 Regression Trees and using eleven preoperative variables as covariates. From this ensemble of trees, we extracted the relative variable importance measure called Total Decrease in Node Impurity (Fig. 1). We observed that only sHE4 and sCA125 out of the 11 variables involved in the analysis show a high relative variable importance, major of 60^22^.

**Figure 2:**
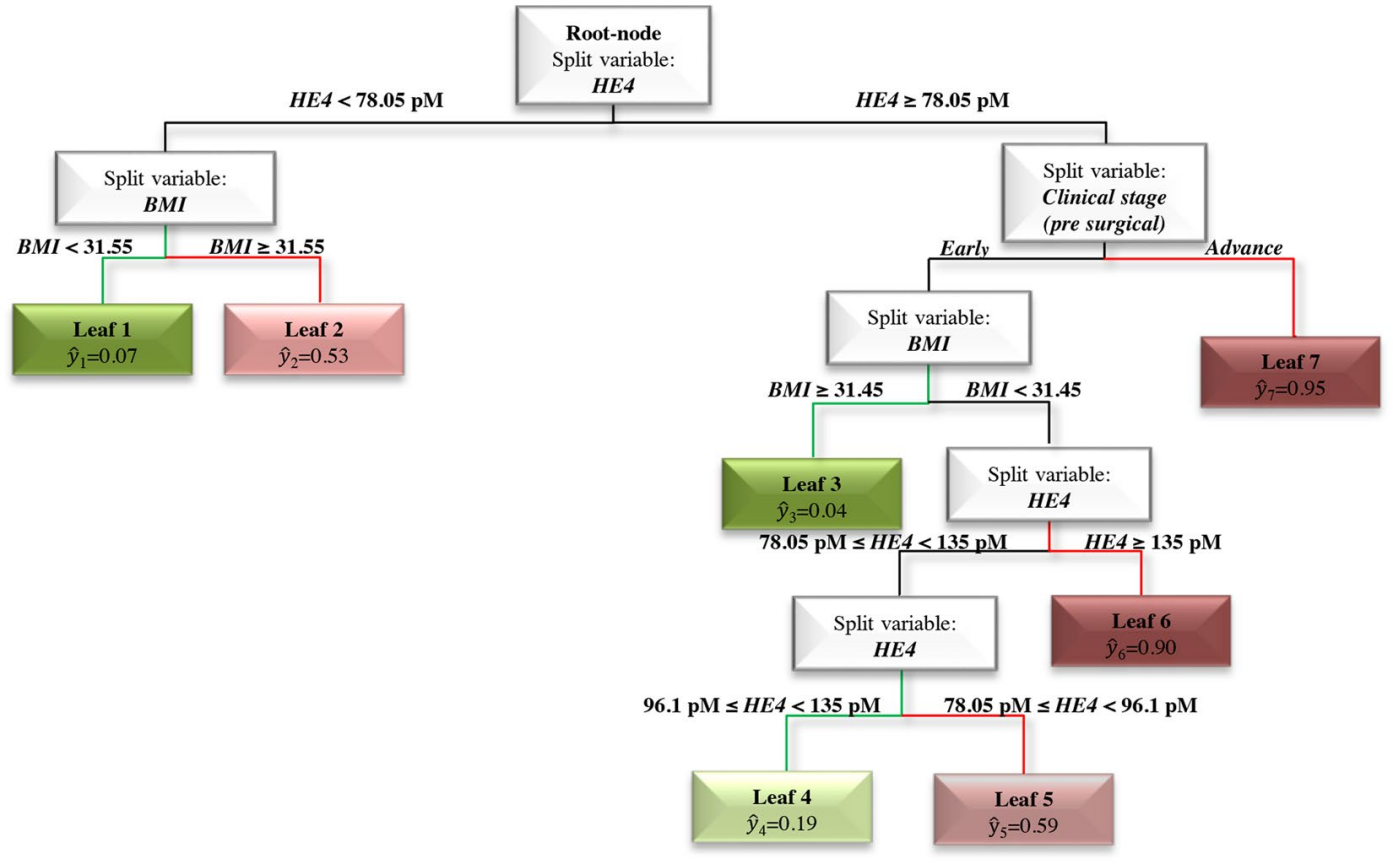
REpresentative Regression Tree (RERT) obtained on all 293 EC patients. In detail, *ŷ* is the relative frequency of patients, clustered within the same final node, having an advanced surgical stage (FIGO stage > I). Low or high values of *ŷ* can be interpreted as low (paths highlighted in green) or high (paths highlighted in red) probability of having a surgical FIGO stage > I, respectively.

Next, after the application of the procedure described in Statistical analysis (see the algorithm), we obtained the *REpresentative Regression Tree* (RERT) reported in Fig. 2. Specifically, sHE4 has the major impact on prediction of the dependent variable (surgical FIGO stage). Looking at the most predictive leafs, we note that patients showing sHE4 ≥ 78.05 pM and advanced clinical stage have a 95% probability of having a surgical FIGO stage > I (see Leaf 7 with ŷ_7_ = 0.95). On the contrary, in the right branch of the tree (sHE4 ≥ 78.05 pM) when clinical stage is early and body mass index (BMI) ≥ 31.45, the probability of having a surgical FIGO stage > I is only 4% (see Leaf 3 with ŷ_3_ = 0.04). At the same level of the tree, we can observe that with BMI < 31.5 and sHE4 ≥ 135 pM the probability of having a surgical FIGO stage > I (see Leaf 6 with ŷ_6_ = 0.90) dramatically increases. Looking at the left branch of the tree (sHE4 < 78.05 pM), women with BMI values < 31.55 show a very low probability of presenting a surgical FIGO stage > I (Leaf 1 with ŷ_1_ = 0.07) (Fig. 2).

When we compare the performance of RERT with the Logistic Regression and the single cross-validated regression tree (RT with CV), our algorithm shows better performance than the two competitors: the AUC is indeed 0.87 against 0.52, shown by Logistic Regression, and 0.75, shown by RT with CV (DeLong’s test, both *p-value* < 0.001) (Table 2, and Table S4 in Supplementary Information, page 4). In addition, RERT outperforms the prediction ability of the single biomarkers sHE4 and sCA125 in terms of AUC (DeLong’s test, both *p-values* < 0.001). Notably, RERT exhibits the highest sensitivity, accuracy, and negative predictive value (NPV) in predicting surgical FIGO stage > I in comparison with all other competitor methods analyzed (Table 2).

**Table 2:** Metrics to assess the performance of the proposed methods evaluated in all 293 EC patients.

Metrics	Clinical Stage	CA125	HE4	RERT with CV	Logistic Regression	RT with CV
ROC-AUC	—	0.69^***^	0.74^**^	0.87	0.52^***^	0.75^**^
Threshold (Youden Index)	—	33.25	81.80	0.28	0.28	0.62
Specificity	0.95	0.82	0.66	0.76	0.66	0.90
Sensitivity	0.36	0.44	0.69	0.90	0.50	0.48
Accuracy	0.75	0.69	0.67	0.81	0.61	0.76
PPV	0.78	0.56	0.51	0.65	0.43	0.72
NPV	0.74	0.74	0.81	0.94	0.72	0.77

**p-values < 0.001, ***p-values < 0.0001: these are *p-values* of the DeLong’s test for the comparison of two AUCs (RERT with CV vs other methods). For major details see Table S4 in Supplementary Tables. RT with CV stands for Regression Tree with Cross-Validation.

The same analyses were performed on a subgroup of 246 patients preoperatively clinically classified as early stage (Fig. S1 in Supplementary Information, page 6). As we observed when exploring the entire cohort of patients, RERT demonstrated the best performance compared to single biomarkers, Logistic Regression and RT with CV, showing the best AUC, sensitivity, accuracy, positive predictive value (PPV) and NPV (Tables S5 and S6 in Supplementary Information, page 4-5).

**Figure 3:**
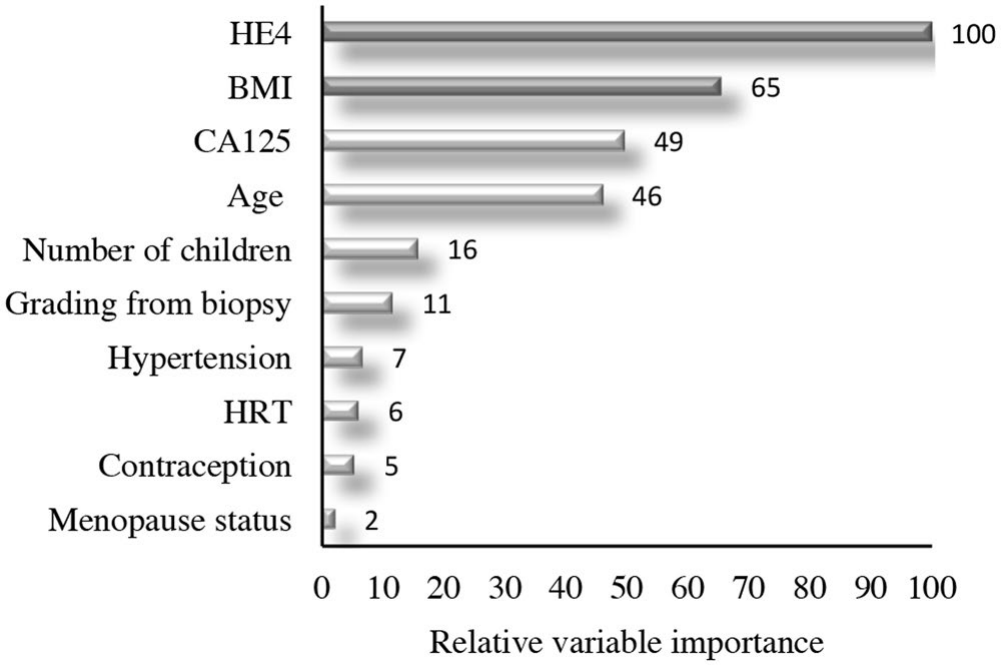
Relative variable importance measure. Total Decrease in Node Impurity- obtained by Random Forests on the subgroup of 219 endometrioid EC patients preoperatively classified as early stage (clinical stage).

**Figure 4:**
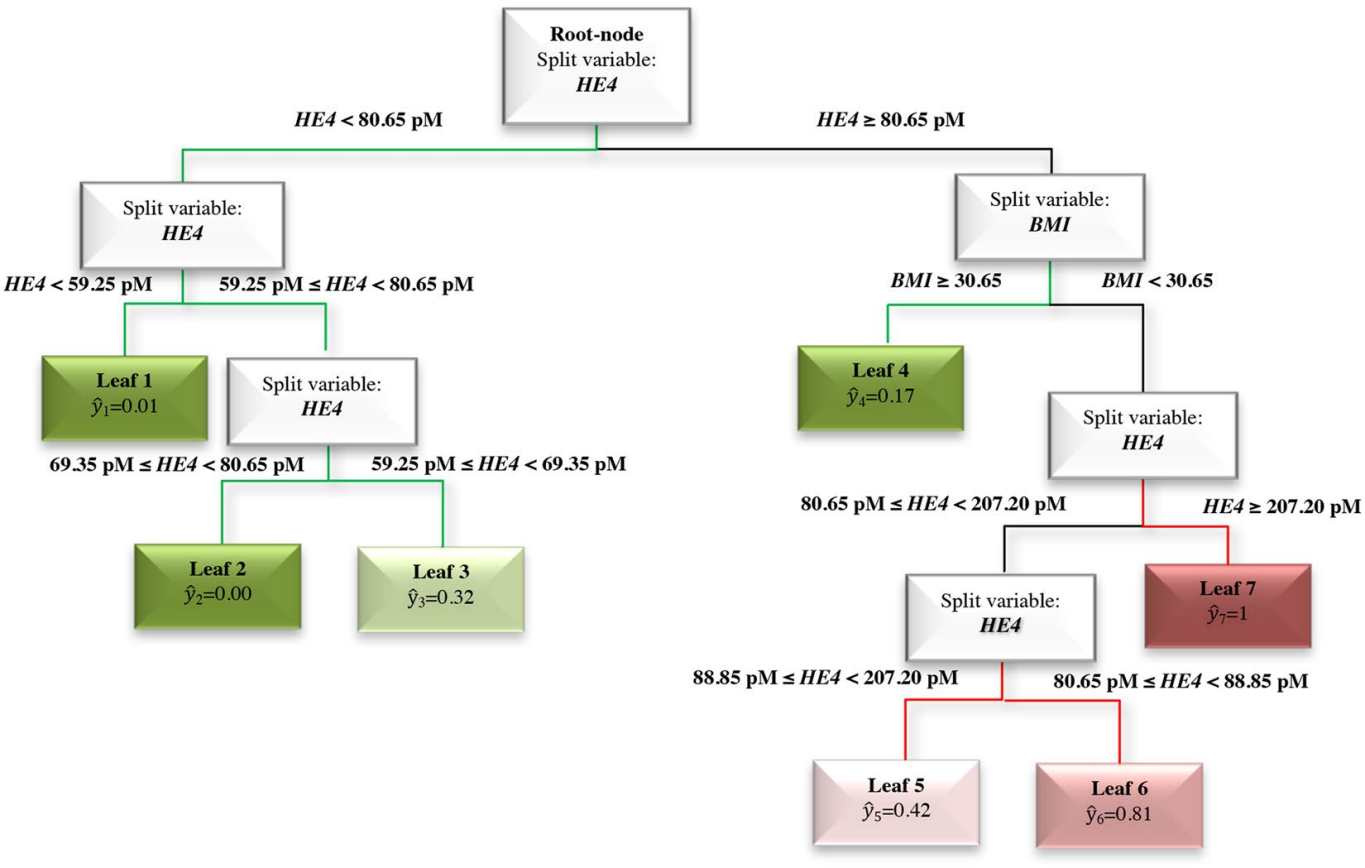
RERT obtained on the the subgroup of 219 endometrioid EC patients preoperatively classified as early stage (clinical stage). In detail, *ŷ* is the relative frequency of patients, clustered within the same final node, having an advanced surgical stage (FIGO stage > I). Low or high values of *ŷ* can be interpreted as low (paths highlighted in green) or high (paths highlighted in red) probability of having a surgical FIGO stage > I, respectively.

Finally, we focused on 219 EC patients preoperatively clinically classified as early stage of endometrioid histotype, since they represent the cohort at higher risk of being surgically undertreated. We ran the Random Forest extracting the relative variable importance measure (Fig. 3) as described above for the whole cohort of patients. In this case, only sHE4 and BMI show a high importance in predicting surgical FIGO stage > I, as confirmed by the RERT (Fig. 4). Looking at the leaves with the lowest and the highest probability of having a surgical FIGO stage > I, we note that the drivers of the analysis are sHE4 and BMI. In fact, when BMI < 30.65 and 80.65 pM ≤ sHE4 < 88.85 pM, the patients have high probability (81%) of having surgical FIGO stage > I (Leaf 6), which increases to 100% when sHE4 >= 207.20 (Leaf 7). On the contrary, when sHE4 is very low (<59.25 pM), the probability drops down to 1% (Leaf 1).

RERT shows the best predictive ability in identifying EC patients with advanced surgical FIGO stage compared to single markers and the other statistical methods analyzed in term of AUC, sensitivity, accuracy, PPV and NPV (Table 3, and Table S7 in Supplementary Information, page 5).

**Table 3:** Metrics to assess the performance of the proposed methods evaluated in 219 endometrioid EC patients preoperatively classified as early stage (clinical stage).

Metrics	CA125	HE4	RERT with CV	Logistic Regression	RT with CV
ROC-AUC	0.58^***^	0.72^*^	0.84	0.57^***^	0.66^**^
Threshold (Youden Index)	13.55	80.70	0.29	0.15	0.18
Specificity	0.39	0.66	0.67	0.76	0.69
Sensitivity	0.76	0.75	0.84	0.41	0.67
Accuracy	0.48	0.67	0.71	0.68	0.68
PPV	0.28	0.39	0.44	0.34	0.40
NPV	0.85	0.89	0.93	0.81	0.87

*p-value = 0.02, **p-value < 0.01, ***p-values < 0.0001: these are *p-values* of the DeLong’s test or Bootstrap test (underlined) for the comparison of two AUCs (RERT with CV vs other methods). For major details see Table S7 in Supplementary Tables. RT with CV stands for Regression Tree with Cross-Validation.

## Discussion

Endometrial cancer is the most common gynecological malignancy in developed countries and endometrioid carcinoma (type I) is the most frequent histological type accounting for 80–90% of EC. A consensus about the surgical management of EC patients has not been reached yet and in particular, the role of pelvic and para-aortic lymphadenectomy is still under debate^23^. However, a correct extent of surgical staging is crucial for the choice of adjuvant treatment and to give indications on patients’ prognosis.

The surgical strategy is usually designed according to the preoperative evaluation that takes into account clinical examination, imaging results, and the pathologic diagnosis of the endometrial biopsy (histotype and grade); this nevertheless leads to frequently underestimate the anatomical tumor spread^8, 24^. The need of developing sensitive and specific tools, able to predict extrauterine spread of cancer, remains a priority in order to avoid undertreatment of high-risk patients or overtreatment of low-risk patients.

In our cohort, 246 EC patients (84%) showed a disease clinically confined to the uterine corpus (clinically early stage). Notably, among these, 63 patients (22%) have been reclassified as FIGO stage > I after surgery. As expected, we found a strong association between clinical and surgical stage, but, in agreement with other research groups^24, 25^, clinical staging was characterized by low sensitivity (36%) and low NPV (74%). The group of patients, erroneously clinically classified as early stage, could benefit from a more reliable preoperative assessment of tumor extension (cervical invasion or extra-uterine disease), based on more sensitive and specific clinical and biological markers than those currently used.

In this study, we applied an original statistical approach for the preoperative prediction of surgical FIGO stage > I based on Regression Trees and related ensemble methods. We used non-parametric tests because they are able to detect complex and non-linear interactions between response variables and qualitative/quantitative covariates, thus proving to be more appropriate for the evaluation of many clinical variables not normally distributed. First of all, parametric tests are unable to fully describe complex data structures in which interactions and non-linearity among covariates are substantial. Secondly, clinicians are generally used to think in terms of low versus high risk of developing a disease, and parametric models, such as multivariate Logistic Regression, while providing the probability of phenomenon occurrence, are silent about the values of indicators signaling different risk clusters. In the current study, we introduced the RERT algorithm conceived to predict preoperatively an advanced surgical FIGO stage using sHE4 and sCA125 biomarkers together with other preoperatively available clinical and pathological variables as covariates (age, BMI, number of children, menopause status, contraception, HRT, hypertension, grading from biopsy, clinical stage). Moreover, through Random Forest^19^, we assessed the relative importance of each variable involved in the analysis, in order to identify the main drivers relevant to predict surgical FIGO stage. The implementation of RERT together with the measures of variable importance is a methodological innovation that we introduced to better understand the contribution of each predictor on FIGO stage jointly with its interaction effect on other variables involved in the analysis. This combination not only enables to determine a small but clinically significant number of variables on which to focus, but also provides an easily interpretable graphical representation of the factors leading to an advanced FIGO stage. As described in the ‘Statistical analysis’ paragraph, the RERT shown more stable and reliable predictions than those offered by a single tree. In particular, we selected the best cross validated Regression Tree among a “black box” of similar trees (“weak learners”) grown on different bootstrap samples and we compared its predictive performance to the performance of sHE4, sCA125, Logistic Regression (frequently used in this framework), and a single cross-validated Regression Tree.

Taking into account the whole series of EC patients, the evaluation of the variable importance showed the highest relative relevance for sHE4 and sCA125. However, while sHE4 maintains its relative importance in RERT, sCA125 loses its impact, as clinically expected. In fact, the vast majority of advanced stage ECs do not spread in the peritoneal cavity, as do type II ECs that represent the minority of cases in this series, in accordance with the epidemiology of this cancer.

RERT showed the best performance in predicting surgical FIGO stage > I compared to serum biomarkers (sHE4, sCA125), to Logistic Regression and to single cross-validated Regression Tree. The sensitivity and the specificity of RERT were 90% and 76%, respectively (NPV = 94% and PPV = 65%) on the entire cohort of EC patients. Considering only clinically early stage EC patients with endometrioid histotype, who represents the cohort at higher risk of being surgically undertreated, the sensitivity and the specificity of RERT were 84% and 67%, respectively (NPV = 93% and PPV = 44%). Our results are not easily comparable with data already published in literature, because to our knowledge, only three groups have focused on finding the best biomarkers’ cutoff for predicting surgical FIGO stage^26–29^. Moore *et al*.^27^, in a prospective study carried out on 124 endometrioid EC patients, identified 70 pM as the best sHE4 cutoff to predict FIGO stages > IA, with a sensitivity and a specificity of 82.1% and 56.1%, respectively (NPV = 82.1%, PPV = 56.1%). Accordingly, in our study, sHE4 levels greater than 81.80 pM (Youden Index cutoff) showed a sensitivity and a specificity of 69% and 66%, respectively (NPV = 81%, PPV = 51%) comparing FIGO stage I vs FIGO stage > I. The slight discrepancy in HE4 performance between the two studies may be due to the different cutoff used and the different comparison between stages. Capriglione *et al*.^28^ assessed sHE4 for the prediction of FIGO stage on 232 EC patients, using the Logistic Regression method. In detail, a cutoff value of 104.3 pM, was able to classify EC patient with FIGO stage II with a sensitivity and specificity of 80.9% and 98.6%, respectively (NPV = 97.3% and PPV = 89.4%) (38), and HE4 showed the best performance reported in the literature up to now, even when a similar cut-off was adopted^29^. In the study of Minar *et al*.^29^, HE4 showed a lower predictive power closer to our results. In that study, both sHE4 and sCA125 determination was performed on 115 endometrioid EC patients and analyzed by the Logistic Regression method^29^. At the cutoff of 110 pM, sHE4 showed a sensitivity and a specificity of 60.9% and 87%, respectively (NPV = 76.9% and PPV = 75.7%), comparing FIGO stage IA vs FIGO stage IB-IV. CA125 showed a lower performance compared to HE4, in agreement with our results.

In several studies, preoperative sHE4 and sCA125 values were correlated with myometrium invasion, tumor grade and cervical invasion^12, 30, 31^ to identify high risk EC patients who may require lymphadenectomy. While the histological grading of the neoplasia is deemed to have major impact in stratifying patients into “risk classes”, we have to highlight its marginal role in classifying patients in early (FIGO I) versus advanced (FIGO > I) stage. In contrast with the literature^12, 30, 31^, in the current analysis its impact on the probability of being surgically staged as advanced (FIGO > I) is very low. Noteworthy, in both the whole and in the endometrioid series, the impact of tumor grade is very lower compared to BMI and age, and a little bit lower compared to number of children.

To conclude, we developed a robust statistical model which is able to preoperatively predict the presence of extrauterine disease in EC patients. RERT provided enhanced predictive value in determining the true advanced FIGO stage, combining serum biomarkers, clinical evaluation (CT imaging) and other preoperative clinic-pathologic characteristics. Such an approach proves to be an effective decisional process regarding the therapeutic options to be performed. More prospective studies in larger cohort of patients are necessary to verify and validate our findings and to translate RERT into clinical practice.

## Materials and Methods

### Study design

The aim of this study is to predict EC patient risk of harbouring an advanced FIGO stage, defined as superior to stage I (>I). To this intent, we used a non-parametric approach belonging to data mining methods, well suited in the case of heterogeneous data and able to deal with relationships amongst variables that are not necessarily linear.

We created a dedicated dataset containing 20 variables reported in Table S1 (in Supplementary Information, page 2). Then, in our analysis, we used a two-step procedure: (*i*) we ran the Random Forest algorithm using the surgical FIGO stage as dependent variable and 11 variables in the dataset as covariates which corresponds to the preoperative clinical characteristics [HE4, CA125, age (in years), BMI, number of children, menopause status, contraception, HRT, hypertension, grading from biopsy, clinical stage (pre-surgical)]. For each of the eleven variables we obtained, through the use of Random Forest, a measure of importance that allowed us to identify the drivers of the surgical FIGO stage prediction. Using the same variables as in the first step, (*ii*) we grew an ensemble of 1000 Regression Trees extracting the most representative pruned tree from the *black-box* obtained. The pruning method permits to avoid the typical overfitting problem of Regression Trees^15^. The final graphic representation gives clinicians a flow-chart with cut-off values enabling a major comprehension of the phenomenon under inspection.

### Patients and samples

Preoperative blood samples from consecutive patients affected by endometrial carcinoma (all Caucasian) treated at the Division of Gynecologic Oncology, Department of Obstetrics and Gynecology, University of Brescia, Italy were obtained from January 2003 to April 2015. The study was performed following the Declaration of Helsinki set of principles and was approved by the Research Review Board-Ethic Committee- of the ASST Spedali Civili, Brescia, Italy (study reference number: NP553). Inclusion criteria for enrollment were: (a) age more than 18 years; (b) biopsy-proven EC; (c) informed consent obtained from the patient. Exclusion criteria were: (a) history of previous or concomitant neoplasm; (b) renal failure; c) preoperative chemo or radiation therapy; (d) not complete surgical staging. All patients underwent total abdominal hysterectomy, bilateral salpingo-oophorectomy, peritoneal washings and pelvic lymph node (with or without paraortic) dissection, following the International Federation of Gynecologists and Obstetricians (FIGO)-International Gynecologic Cancer Society (IGCS) Clinical Practice Guidelines^32^. EC patients were staged according to the FIGO 2009 staging system, whilst the histological classification followed WHO criteria.

Fasting blood samples were collected the day before surgery. Serum was separated by centrifugation at 1500 g for 10 minutes within 1 hour, frozen in liquid nitrogen and then stored at –80°C until analysis. EC patients’ charts were reviewed and all the clinico-pathological characteristics were collected in a dedicated data set (Table 1). Pre-surgical clinical stage was assessed on the basis of clinical evaluation and imaging by expert Gynecologic Oncologists.

### HE4 and CA125 serum concentrations measurements

Preoperative sHE4 concentrations were measured using the CMIA assay (Abbott Diagnostics Division, Wiesbaden, Germany) on the fully automated Architect instrument (Abbott Diagnostics Division), as previously reported by our group^33^. The dynamic range of sHE4 detection goes from 20 to 1500 pM with an automated 1:10 dilution protocol that extends the linear range up to 15,000 pM. The intra-assay and total imprecision (CV %) of the CMIA sHE4 assay ranged from 2.11 to 2.93% and from 3.13 to 3.70%, depending on the concentrations of the assays’ positive controls^33^. Preoperative sCA125 values were also determined by CMIA assay (Abbott Diagnostics Division) on Architect. The assays is linear up to 1000 U/mL and has a normality threshold at 35 U/mL^34^. All the assays were carried out at the Laboratory Analysis, ASST Spedali Civili of Brescia, Italy, following the manufacturer’s recommendations.

### Statistical analysis

In our empirical analyses, we adopted the Random Forest^19^ procedure with relative variable importance metrics and Regression Trees^15, 35^ with the end to identify the most important variables and then provide accurate predictions of the patients’ surgical FIGO stage. To overcome the intrinsic instability of the Regression Trees (“small changes in the data lead to big changes in the results”), we proposed a new procedure which stabilizes the results across multiple replications. Specifically: (*i*) we drew 1000 bootstrap samples from the original data set, stratifying according to histotype; (*ii*) in each sample we grew a tree, and (*iii*) among these 1000 different trees, we selected the best performer in terms of Area Under the Curve (AUC) of the Receiver Operator Characteristic (ROC) curve: this tree is our *REpresentative Regression Tree* (RERT; see below for technical details).
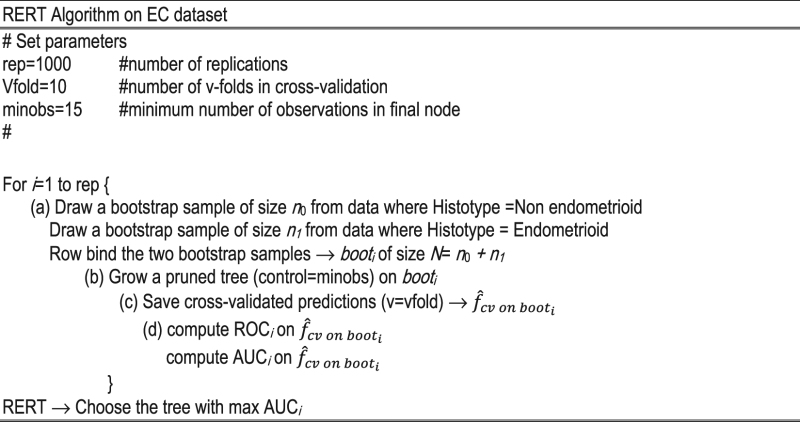


The rational of this procedure is very simple. In the empirical analyses on our dataset, we noted that among the 1000 trees generated on the corresponding 1000 bootstrap samples, those with the best performance in term of AUC were very similar. In other terms, trees with high AUC (>0.80) have chosen the same variables at same depth, and the cut-off points were very close each other. Choosing the tree with the best performance means finding the leader of a subset of similar trees. All the additional analysis on the dataset are available upon request.

Since the low data set dimension (293 patients) does not guarantee an efficient partition between training and test sets, we adopted the *v-fold cross–validation* (where *v*, for convention, is equals to 10) using the complete data set thus obtaining reliable results.

RERT follows the idea that from an ensemble algorithm of trees (“black box”) it is possible to extract a simple, interpretable and accurate model^36–39^. In other terms, RERT could be a possible solution to the eternal conflict between accuracy and simplicity (interpretability)^40^. For major details on Regression Tree and Random Forest see Supplementary Methods (Supplementary Information, from page 7 to 11).

The performance of RERT, in terms of AUC, specificity, sensitivity, accuracy, computed in correspondence of the Youden Index was compared to the performances of: (*i*) sCA125; (*ii*) sHE4; (*iii*) Logistic Regression; (*iv*) single cross-validated Regression Tree (RT with CV). Moreover, the ROC-AUCs were compared two by two by the DeLong test^41^.

All these analyses were performed on: (*i*) all the cohort of EC patients; (*ii*) the subsample of the early clinical stage patients; (*iii*) the subsample of the early clinical stage patients with endometrioid histological type. Furthermore, the association between the concentrations of sHE4 or sCA125 and clinicopathologic parameters was investigated using either the Wilcoxon-Mann-Whitney test or the Kruskal-Wallis test (considering *p-value* < 0.05 as significant). Finally, the association between preoperatively-available *quantitative* and *qualitative* variables and surgical FIGO stage were investigated by means of Wilcoxon-Mann-Whitney and Pearson’s Chi-squared tests, respectively.

All statistical analysis was performed using R 3.2.0 (R Development Core Team, 2010).

## Electronic supplementary material

Supplementary informations
