# Supplementary material for: RERT: A Novel Regression Tree Approach to Predict Extrauterine Disease in Endometrial Carcinoma Patients
Source: Sci Rep. 2017 Sep 5;7:10528. doi: 10.1038/s41598-017-11104-4 (PMC5585365; doi:10.1038/s41598-017-11104-4)
Supplement: Supplementary file 1 — Supplementary informations [file 41598_2017_11104_MOESM1_ESM.pdf]

SUPPLEMENTARY INFORMATION

RERT: A NOVEL REGRESSION TREE APPROACH TO PREDICT EXTRAUTERINE DISEASE IN ENDOMETRIAL CARCINOMA PATIENTS

Marika Vezzoli[1§], Antonella Ravaggi[2,§*], Laura Zanotti[2], Rebecca Angelica Miscioscia[3], Eliana Bignotti[4], Monica Ragnoli[4], Angela Gambino[3], Giuseppina Ruggeri[5], Stefano Calza[1,6], Enrico Sartori[3], Franco Odicino[3]

[1] Department of Molecular and Translational Medicine, Unit of Biostatistics, University of Brescia, Brescia, Italy.

[2] "Angelo Nocivelli" Institute of Molecular Medicine, Division of Obstetrics and Gynecology, University of Brescia, Brescia, Italy.

[3] Department of Obstetrics and Gynecology, University of Brescia, Brescia, Italy.

[4] Division of Obstetrics and Gynecology, ASST Spedali Civili of Brescia, Brescia, Italy.

[5] Laboratory Analysis, ASST Spedali Civili of Brescia, Brescia, Italy.

[6] Department of Medical Epidemiology and Biostatistics, Karolinska Institutet, Stockholm, Sweden.

Supplementary Tables

Table S1. Variables contained in the dataset

Variable	Type	Code (in bold) attributed to each category
**FIGO stage**	dummy variable* - Dependent variable (*y*)	I → **0**<br>> I → **1**
**HE4**	quantitative variable - Covariate	-
**CA125**	quantitative variable - Covariate	-
**Age (years)**	quantitative variable - Covariate	-
**BMI**	quantitative variable - Covariate	-
**Number of children**	quantitative variable - Covariate	-
**Menopause status**	dummy variable - Covariate	No → **0**<br>Yes → **1**
**Contraception**	dummy variable - Covariate	No → **0**<br>Yes → **1**
**HRT**	dummy variable - Covariate	No → **0**<br>Yes → **1**
**Hypertension**	dummy variable - Covariate	No → **0**<br>Yes → **1**
**Grading from biopsy**	categorical variable - Covariate	Hyperplasia → **0**<br>G1 → **1**<br>G2 → **2**<br>G3 → **3**
**Clinical stage (pre-surgical)**	dummy variable - Covariate	Early (FIGO ≤ I) → **0**<br>Advanced (FIGO>I) → **1**
**Histotype**	dummy variable	Non endometrioid → **0**<br>Endometrioid → **1**
**Surgical Grading**	categorical variable	G1 → **1**<br>G2 → **2**<br>G3 → **3**
**Myometrial invasion**	categorical variable	M0 → **1**<br>M1 → **2**<br>M2 → **3**
**Extension to cervix**	dummy variable	No → **0**<br>Yes → **1**
**Ovarian metastases**	dummy variable	No → **0**<br>Yes → **1**
**Lymph nodes status**	dummy variable	Negative → **0**<br>Positive → **1**
**Lymphovascular invasion**	dummy variable	Absent → **0**<br>Present → **1**
**Positive peritoneal cytology**	dummy variable	No → **0**<br>Yes → **1**

*Dummy variables means a variable that assumes only two values (e.g. 0 or 1)
BMI = Body Mass Index; **HRT** = Hormone Replacement Therapy

Table S2. Association between preoperatively-available quantitative variables and surgical FIGO stage (I vs >I)

Variables	Surgical FIGO stage						
	Median			Range		95th perc	
	I	>I	*p-value*	I	>I	I	>I
**HE4 (pmol/L)**	66.10	107.40	***1.84E-11***	6.50 - 346.70	34.50 - 653.00	197.40	526.60
**CA125 (U/ml)**	15.90	25.00	***6.26E-06***	2.40 - 238.00	3.90 - 2922	58.12	299.90
**Age (years)**	64.50	65.00	*0.1372*	32.00 - 82.00	28.00 - 87.00	80.00	82.10
**BMI**	27.10	25.50	*0.1927*	14.00 - 47.83	16.70 - 44.90	37.48	37.26
**Number of children**	2.00	2.00	*0.6455*	0.00 - 8.00	0.00 - 6.00	4.00	3.10

p-values were computed using non-parametric Wilcoxon-Mann-Whitney test. Missing values are not considered in the test procedure. In bold *p-values*<0.05

Table S3. Association between preoperatively-available qualitative variables and surgical FIGO stage (I vs >I)

Variables	Surgical FIGO stage		
	n. (%)		
	I	>I	*p-value*
**Menopause status**			
No	27 (9%)	8 (3%)	
Yes	167 (57%)	90 (31%)	*0.1529*
**Contraception**			
No	125 (61%)	68 (33%)	
Yes	8 (4%)	5 (2%)	*0.8138*
**HRT**			
No	148 (56%)	75 (28%)	
Yes	27 (10%)	14 (6%)	*0.9490*
**Hypertension**			
No	95 (32%)	48 (16%)	
Yes	99 (34%)	51 (18%)	*0.9375*
**Grading from biopsy**			
Hyperplasia	6 (2%)	0 (0%)	
G1	65 (24%)	17 (6%)	
G2	57 (21%)	34 (13%)	
G3	52 (20%)	35 (14%)	***0.0096****
**Clinical stage (pre-surgical)**			
Early	183 (63%)	63 (22%)	
Advance	9 (3%)	35 (12%)	***3.25E-12***

p-values were computed using Pearson's Chi-squared test. In bold *p-values*<0.05

Table S4. *P-values* of the DeLong's test for the comparison of two AUC (symmetric matrix). The AUCs were computed considering all 293 EC patients.

	**CA125**	**HE4**	**RERT with CV**	**Logistic Regression**	**RT with CV**
**CA125**	-				
**HE4**	***0.0360***	-			
**RERT with CV**	***5.79E-07***	***0.0004***	-		
**Logistic Regression**	***0.0218***	***0.0002***	***4.84E-10***	-	
**RT with CV**	***0.0452***	*0.8567*	***0.0009***	***0.0002***	-

In bold *p-values*<0.05. RT with CV stands for Regression Tree with Cross-Validation

Table S5. Metrics to assess the performance of the proposed methods evaluated in 246 EC patients clinically (pre-surgery) classified as early stage.

**Metrics**	**CA125**	**HE4**	**RERT with CV**	**Logistic Regression**	**RT with CV**
**ROC-AUC**	0.60***	0.72**	0.86	0.50$^{\underline{***}}$	0.67***
**Threshold (Youden Index)**	13.55	81.80	0.28	0.14	0.21
**Specificity**	0.40	0.66	0.82	0.82	0.54
**Sensitivity**	0.78	0.70	0.83	0.38	0.76
**Accuracy**	0.50	0.67	0.82	0.71	0.60
**PPV**	0.31	0.42	0.61	0.42	0.36
**NPV**	0.84	0.86	0.93	0.79	0.87

** *p-value*=0.003, *** *p-values*<0.0001: *p-values* of the DeLong's test or Bootstrap test (underlined) for the comparison of two AUCs (RERT with CV vs other methods). For major details see Supplementary Table S6. RT with CV stands for Regression Tree with Cross-Validation

Table S6. *P-values* of the DeLong's test or Bootstrap test* for the comparison of two AUC (symmetric matrix). The AUCs were calculated considering 246 EC patients clinically (pre-surgery) classified as early stage.

	CA125	HE4	RERT with CV	Logistic Regression	RT with CV
**CA125**	-				
**HE4**	***0.0104***	-			
**RERT with CV**	***4.85e-07***	***0.0030***	-		
**Logistic Regression**	*0.1714**	***0.0020****	***8.49e-08****	-	
**RT with CV**	*0.1618*	*0.0577*	***7.38e-05***	***0.0253****	-

In bold *p-values<0.05*. RT with CV stands for Regression Tree with Cross-Validation

Table S7. *P-values* of the DeLong's test or Bootstrap test* for the comparison of two AUC (symmetric matrix). The AUCs were calculated considering 219 EC patients preoperatively classify as early stage (clinical stage) and with endometrioid histotype.

	CA125	HE4	RERT with CV	Logistic Regression	RT with CV
**CA125**	-				
**HE4**	***0.0047***	-			
**RERT with CV**	***4.14e-06***	***0.0203***	-		
**Logistic Regression**	*0.9130**	***0.0385****	***9.65e-05****	-	
**RT with CV**	*0.0584*	*0.0584*	***0.0014***	*0.2287**	-

In bold *p-values<0.05*. RT with CV stands for Regression Tree with Cross-Validation

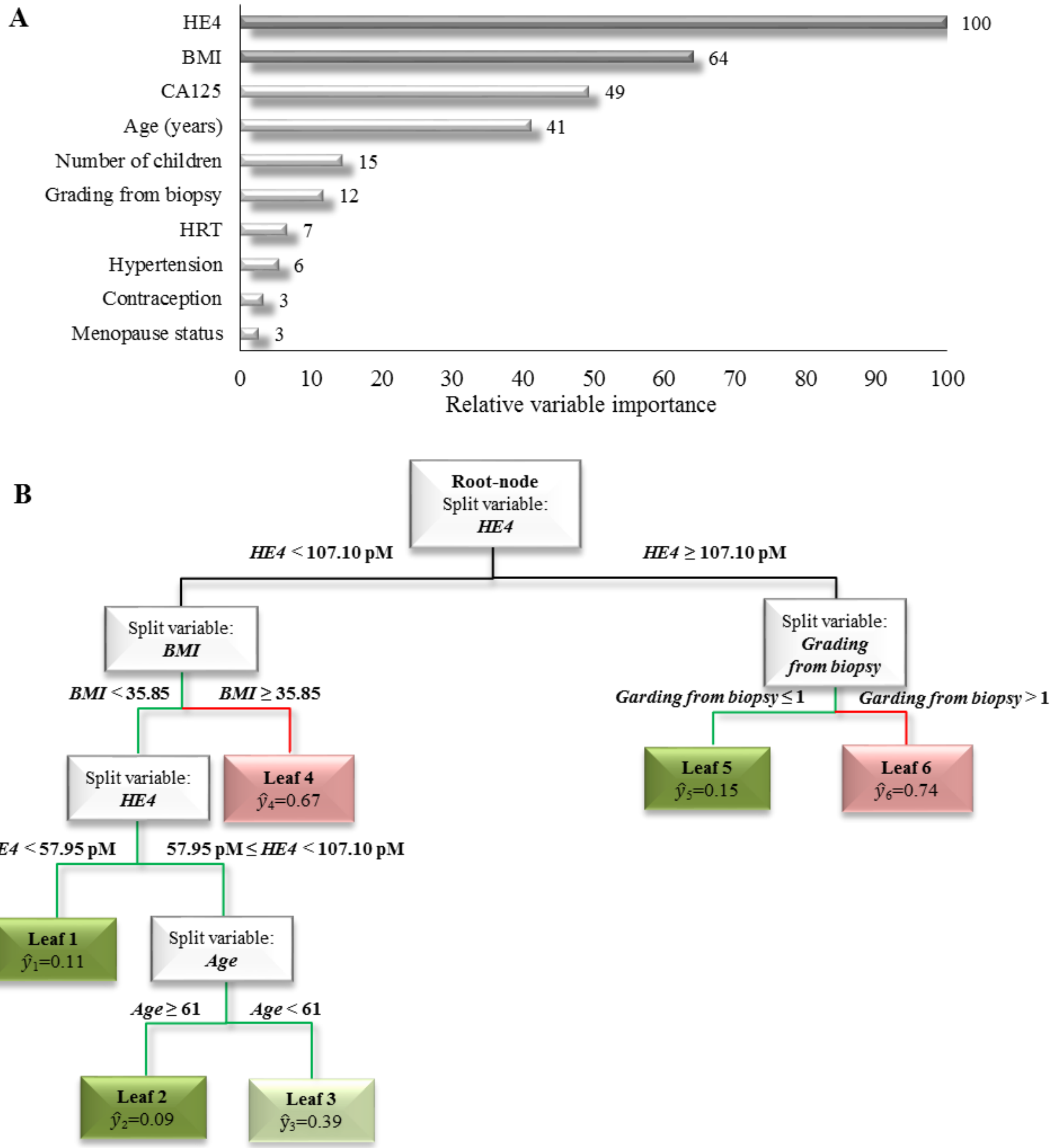

Figure S1. Variable importance measure obtained by Random Forests considering 246 patients preoperatively clinically classified as early stage (**A**) and RERT on the same cohort (**B**). In detail, ŷ is the relative frequency of patients, clustered within the same final node, having an advanced surgical stage (FIGO stage >I). Low or high values of ŷ can be interpreted as low (paths highlighted in green) or high (paths highlighted in red) probability of having a surgical FIGO stage >I, respectively.

Supplementary Methods

Regression Tree

Regression Tree[1] is a non-parametric method[a] that recursively partitions the predictor space into disjoint and homogeneous regions (called nodes or leaves) with respect to the response variable Y by means of a series of subsequent splits. At each step, the algorithm chooses the variable that best divides the data in homogeneous subgroups having the objective to minimize the prediction error within each node, finally computing predictions as the average of the Y values within each terminal nodes. The estimation process is based on the v-fold cross-validation[2], through which the data are partitioned into v equally (or nearly equally) sized folds, next running v interactions of training and validation in such an extent that within each interaction a different fold of the data is held-out for validation, while the remaining (v – 1) folds are used for learning.

In order to avoid the problem of overfitting[b], Regression Tree uses the pruning technique as a stopping criteria, by growing a large tree and then prune it back until it reaches an optimal size. In doing so, the algorithm uses a cost-complexity parameter criterion that balances the size of the tree with the goodness of fit by finding the "right" compromise between simplicity and efficiency of the model.

One of the main advantage of Regression Trees is the interpretability of their results: the output provides a visual representation in which each observation "travels" from the root node (the node containing the entire training set) to one of the leaves where the prediction is made. The result is a series of *Rules of Thumb* where the algorithm automatically identifies the most important variables and corresponding thresholds useful, for example, for clinicians during a decision process.

[a] It does not require assumption on the distribution of the dependent variable Y.

[b] Overfitting problems arise when the model is excessively complex, having too many parameters relative to the number of observations used in the analysis. A model in overfitting provides bad predictions which are extremely sensitive to small perturbations in the data.

Technical issues on Regression Trees

Let $\mathbf{X} = [(X_1, \ldots, X_r)]$ be a collection of *r* vectors of predictors, both quantitative and qualitative. Let *T* denote a tree with $m = 1, \ldots, M$ terminal nodes, i.e. the disjoint regions $\tilde{T}_m$, and by $\Theta = \theta_1, \ldots, \theta_m$ the parameter that associates each m-th θ value with the corresponding node. A generic dependent variable *Y* conditional on Θ assumes the following distribution

$$f(y_i|\Theta) = \sum_{m=1}^{M} \theta_m I(\mathbf{X} \in \tilde{T}_m)$$

where θ_m represents a specific $\tilde{T}_m$ region and *I* denotes the indicator function that takes the value of 1 if $\mathbf{X} \in \tilde{T}_m$, 0 otherwise. This signifies that predictions are computed by the average of the *Y* values within the terminal nodes, i.e.

$$\hat{y}_i = \hat{\theta}_m \Rightarrow \frac{1}{N_m} \sum\nolimits_{\mathbf{x}_i \in \tilde{\boldsymbol{T}}_{\boldsymbol{m}}} y_i$$

with $i = 1, \ldots, N$ the total number of observations and N_m the number within the *m*-th region.

Computationally, the general problem for finding an optimal tree is solved by minimising the following loss function

$$\underset{\Xi=\{T,\Theta\}}{\operatorname{argmin}} L = [Y - f(Y|\Theta)]^2.$$

This entails selecting the optimal number of regions and corresponding splitting values.

Let s^* be the best split value and $R(m) = N_m^{-1} \sum_{\mathbf{x}_i \in \tilde{T}_m} (y_i - \hat{c}_m)^2$ be the measure of the variability within each node, where $\hat{c}_m$ is the average of y_i's within the *m*-th node. Thus, the fitting criterion is given by

$$\Delta R(s^*, m) = \max_{s^*} \Delta R(s, m)$$

with

$$\Delta R(s, m) = R(m) - [R(m_1) + R(m_2)].$$

It is important to note that the optimization is local. It means that in the greedy methods there is no assurance that successive locally optimal decisions lead to the global optimum[3]. Moreover, $R(T) = \sum_{m \in \tilde{\tilde{T}}} R(m)$, is the loss function of the entire tree, where $\tilde{\tilde{T}}$ is the set of its terminal nodes.

Having found the best split s^*, the data are partitioned into two regions and the splitting process is repeated on each of them. This procedure can be carry until when in each leaves there is only 1 case; in this way, we are in presence of overfitting and the tree, denoted T_{max}, is not a good predictor. Hence, an important issue is the choose of the tree size.

Breiman and Stone[4] proposed a method called pruning, based on the cross-validation. The idea is to choose subtrees using the loss function $R(T)$, adjusted by a complexity parameter $\alpha \geq 0$:

$$R_\alpha(T) = R(T) + \alpha \left|\tilde{\tilde{T}}\right|$$

where $|\tilde{\tilde{T}}|$ denotes the number of terminal nodes in T. The idea is to find, for each α, the subtree $T_\alpha \subset T_{max}$, where T_{max} denotes the tree goes in overfitting, obtained by pruning T_{max} in order to minimize $R_\alpha(T)$. The parameter of cost complexity α governs the trade- off between the size and its goodness of fit to the data. Large values of α result in smaller trees, and conversely for smaller values of α.

Random Forest

One of the major complaints of tree-based model is their instability. Small changes in the predictor distribution can drastically change the structure of the resulting tree. A consequence of unstable methods is that the prediction error is high.

An approach that mitigates this problem and increases the accuracy of the predictors consists of developing a population of simple models, called base or weak learner (in our case trees), within the perturbed training set and combining them in order to form a composite predictor (see Figure S.M.1 for a graphical representation of how these algorithms work). These models, known as ensemble learning, include, among others, Bagging[5], Boosting[6] and Random Forest[7]. The last one, repeatedly used in this paper, have become increasingly popular in medicine, genetics and in neurosciences.

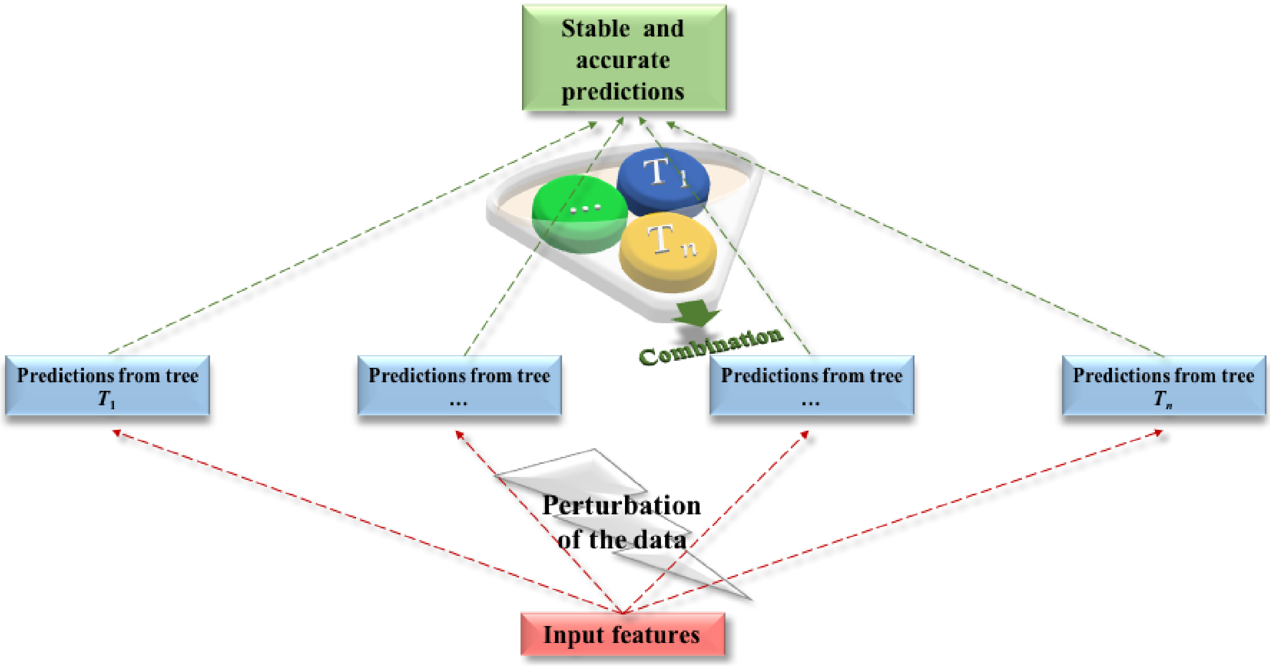

Figure S.M.1: Graphical representation of how ensemble algorithms work

The procedure used by Random Forest for combining different trees and obtaining accurate predictions are reported below.

Random Forest Algorithm - Regression

Set parameters
BOOT #number of replications
n_{min} #identify a minimum node size
g #number of variables selected by the algorithm at each node of the tree
#

For *i*=1 to *BOOT* {
(a) Draw a bootstrap sample $boot_i$ of size *N* from the training data
(b) Grow a tree T_{booti} to the bootstrapped data, by recursively repeating the following steps for each node of the tree, until the minimum node size n_{min} is reached.
(*i*) Select *g* variables at random from the *r* covariates
(*ii*) Take the best split/variable among the *g* variables available
(*iii*) Split the node in two child nodes.
}
From the ensemble of trees, the prediction at a new point *x* is:

$$\hat{f}_{rf}^{B}(x) = \frac{1}{BOOT} \sum_{i=1}^{BOOT} T_i(x)$$

From Random Forest is possible to extract two variable importance measures which identify the covariates that have a major impact on the prediction of the response variable. In this paper we consider only one of them, the Total Decrease in Node Impurity (known also with the name of Gini

Importance). For evaluating the discriminatory power of a variable, this measure accumulates the Gini gain over all splits of trees grown in the forest[8].

In detail, at each tree of Random Forest, the heterogeneity reductions due to variable X_r over the set of nonterminal nodes are summed up and the importance of X_r is computed averaging the results over all the trees of the ensemble. Formally, let $d_{rm^c}^{T_i}$ be the decrease in the heterogeneity index due to X_r at the nonterminal node $m^c \in M^c$ of the T_i tree. The variable importance of r-th variable over all the trees is:

$$\widehat{VI}_{X_r} = \frac{1}{BOOT} \sum_{i=1}^{BOOT} \sum_{m^c \in M^c} d_{rm^c}^{T_i} I_{rm^c}^{T_i}$$

where $I_{rm^c}^{T_i}$ is the indicator function which equals 1 if the r-th variable is used to split node m^c and 0 otherwise.

References

1. Breiman L, Friedman J, Olshen R, Stone C. Classification and Regression Trees. Wadsworth Inc., California, 1984.
2. Stone M. Asymptotics for and against cross-validation. Biometrika 1977;64:29–35.
3. Duda, RO, Hart, PE, Stork, DG. Pattern classification. Wiley & Sons, USA, 2006.
4. Breiman, L, Stone, CJ. Parsimonious binary classification trees. Santa Monica, California, Technology Service Corporation, Technical report,1978.
5. Breiman L. Bagging predictors. Mach Learn 1996;26:123–140.
6. Friedman JH. Greedy function approximation: a gradient boosting machine. Ann Stat 2001;29:1189–1232.
7. Breiman L. Random forests. Mach Learn 2001;45:5–32.
8. Hastie, T, Tibshirani, R, Friedman J. The elements of statistical learning: data mining, inference, and prediction. Springer, New York, 2001.
